# Editorial: Rising stars in cellular neuropathology 2022

**DOI:** 10.3389/fncel.2024.1383629

**Published:** 2024-03-06

**Authors:** Christian Zammit, Jiamei Lian, Mario Valentino

**Affiliations:** ^1^Department of Anatomy, University of Malta, Msida, Malta; ^2^Antipsychotic Research Laboratory, School of Medical, Indigenous and Health Sciences and Molecular Horizons, University of Wollongong, Wollongong, NSW, Australia; ^3^Department of Physiology and Biochemistry, University of Malta, Msida, Malta

**Keywords:** human induced dorsal forebrain precursors, α-Synuclein, calcium signaling, creatine, photoreceptors, Degenerative Cervical Myelopathy

We are grateful to all authors and reviewers for their outstanding contributions to this Research Topic. This compilation of original research articles aims to shed light on clinical and basic issues in cellular neuropathology, and provide the basis for potential therapeutic intervention.

Vidal's group presents two research articles on Degenerative Cervical Myelopathy (DCM). This involves spinal cord dysfunction from compression of the vertebral column around the neck region resulting from age-related changes that also cause pain, gastrointestinal dysfunction and severe neurological deficits. Using a mouse model of DCM, Farkas et al. illustrate the alterations of gut microbiota using 16S rRNA-based detection of bacterial groups from fecal samples in parallel to the altered response in immune cell composition by flow cytometry. The study reports that DCM may cause dimorphic gut dysbiosis with more pronounced effects in males. Specific bacterial families, such as Lachnospiraceae and Muribaculaceae, were significantly altered. These changes were linked to variations in microbe-derived metabolic products. This study highlights a reduction in butyrate-producing bacteria and a lesser impact on lactate-producing bacteria. Monitoring butyrate levels is valuable, yet detecting short-chain fatty acids in clinical settings remains challenging due to their volatility.

Ojeda et al. present a highly detailed analysis of structural and molecular alterations in the spinal cord, neuronal populations, neuromuscular junctions, muscle fibers, and gut that occurs in the DCM model. The outcome shows a significant reduction in locomotion and muscle strength, as well as a shift in gut microbiota. This compliments their previous study that shows that DCM causes gut dysbiosis. These findings not only address possible mechanisms linking the maintenance of cells housed in the spinal cord, but the influence of gut microbiota, and its derived metabolites. This is the first study that investigates the neuromuscular axis after DCM, and sheds light about the potential mechanism in sustained muscle fatigue in patients.

McCaughey-Chapman et al. assess the maintenance of aging factors in a new protocol utilizing stable, non-immunogenic chemically modified mRNA to convert adult human dermal fibroblasts to human induced dorsal forebrain precursor (hiDFP) cells, which can subsequently differentiate into cortical neurons. The authors focus on aging markers, genes, senescence, reactive oxygen species, and DNA methylation in donors and provide data comparing the donor fibroblasts, hiDFPs and induced pluripotent stem cells (iPSCs). In summary, in comparison to the direct conversion to neurons, the current progenitors are expandable, and provide a platform for drug testing and high-throughput disease modeling. The authors further perform assays to examine telomere length, mitochondrial activity and expression of genes known to be changed in aging. This study underscores the importance of incorporating aging traits into hiDFNs when modeling neurodegenerative diseases to ensure translational relevance.

The involvement of α-Synuclein (αSYN) in Parkinson's disease is multifaceted but is primarily attributed to the toxic oligomeric protofibrils that disrupt cellular homeostasis. Tomagra et al. examined the impact on network functionality during development following the exogenous application of αSYN on cultured mouse embryo neurons isolated from the substantia nigra and grown on microelectrode arrays (MEAs). Results from this study show that αSYN attenuates the firing of dopaminergic neurons but only within a specific time window of days *in vitro*. The authors hypothesize that the spontaneous firing discharge are linked to an initial loss of neurons and a deficiency in the release of dopamine in the striatum which may result in subsequent motor impairment. Although the precise molecular mechanisms remains unclear, targeting the detrimental functions arising from its dysregulation could unveil innovative therapeutic approaches.

Calcium signaling is considered a substrate for glial excitability. The study by Lawson et al. used a novel model of acute and chronic nociception for the non-invasive detection of oscillating calcium transients in mono- and co-cultures of iPSC astrocytes as well as iPSC sensory neuron-astrocyte co-cultures utilizing electrical stimulation and MEAs. Their approach stems from the observation that targeting nervous system pathologies should include the phenotypic effects on astrocytes and thus, the calcium transients could serve to implement current screening methods based on detecting neuronal electrical activity. The authors convincingly demonstrate that both neurons and astrocytes in culture could be selectively characterized in real time. This is important as it can provide a phenotypic readout in human cell assays, and increase the ability to identify diverse drug candidates and forecast their clinical effectiveness.

In the study by Ferdous et al. the authors provide evidence, using Chx10-Cre Lsd1^fl/fl^ mice and Rho-iCre75 Lsd1^fl/fl^ mice, that Lysine specific demethylase-1 (*Lsd1*) plays an important role in early retinal development as deletion of *Lsd1* leads to a loss of retinal function and morphological defects in the Chx10-Cre line. Interestingly, the specific deletion of *Lsd1* in photoreceptors using iRho-Cre did not alter retinal function and histology. Their analysis involved a combination of *in vivo* methods (ERG, SD-OCT) and postmortem analysis through histology, immunofluorescence and EM. They demonstrate that loss of *Lsd1* in retinal progenitor cells but not in adult rod photoreceptor cells leads to functional and structural defects. The current study is however limited to only one timepoint (P30) that coincides after retinal development is complete in the Chx10-Cre Lsd1^fl/fl^ mice. Thus, a more complete picture of the model is warranted in future studies.

Tran et al. investigated the effects of creatine prophylactic supplementation on the developing fetal sheep brain following a brief period of acute hypoxia. Using RT-qPCR and IHC, the authors quantified the effects of gene regulation and the pathological responses on multiple brain regions. The potential neuroprotective effects of creatine, administered before and during the hypoxic event, are also investigated. This study shows that the cortical gray matter, thalamus and hippocampus are susceptible to pathology irrespective of creatine treatment, with involvement of cell death and neuroinflammation. Changes in expression of many genes are also reported but with minimal protection except for attenuation of astrogliosis in the corpus callosum. Of interest, creatine induced alteration in gene regulation in gray matter with evidence of increased myelination within the sub cortical white matter. The increase in myelin maturation may impact sensory information processing with dire consequences in neurodevelopment.

In conclusion, this Research Topic presents a compilation of high-quality research articles by early-stage researchers, offering significant insights into various aspects of cellular neuropathology. Overall, these findings underscore the complexity of neuropathological conditions and advocate for multidisciplinary approaches in treatment options.

## Author contributions

CZ: Writing—review & editing. JL: Writing—review & editing. MV: Writing—original draft, Writing—review & editing.

